# Sequential adaptive strategies for sampling rare clustered populations

**DOI:** 10.1007/s10260-023-00707-z

**Published:** 2023-06-13

**Authors:** Fulvia Mecatti, Charalambos Sismanidis, Emanuela Furfaro, Pier Luigi Conti

**Affiliations:** 1 University of Milano-Bicocca, U7 Via Bicocca degli Arcimboldi 8, 20126 Milano, Italy; 2 Global Tuberculosis Programme, WHO, Geneva, Switzerland; 3 Università Cattolica del Sacro Cuore, Milano and University of California, Davis, CA USA; 4 Sapienza Università di Roma, Roma, Italy

**Keywords:** Over-sampling, Poisson sampling, Informative designs, Pseudo Horvitz-Thompson estimator, Asymptotics, Budget and logistic constraints, Intra-cluster variation

## Abstract

A new class of sampling strategies is proposed that can be applied to population-based surveys targeting a rare trait that is unevenly spread over an area of interest. Our proposal is characterised by the ability to tailor the data collection to specific features and challenges of the survey at hand. It is based on integrating an adaptive component into a sequential selection, which aims both to intensify the detection of positive cases, upon exploiting the spatial clustering, and to provide a flexible framework to manage logistics and budget constraints. A class of estimators is also proposed to account for the selection bias, that are proved unbiased for the population mean (prevalence) as well as consistent and asymptotically Normal distributed. Unbiased variance estimation is also provided. A ready-to-implement weighting system is developed for estimation purposes. Two special strategies included in the proposed class are presented, that are based on the Poisson sampling and proved more efficient. The selection of primary sampling units is also illustrated for tuberculosis prevalence surveys, which are recommended in many countries and supported by the World Health Organisation as an emblematic example of the need for an improved sampling design. Simulation results are given in the tuberculosis application to illustrate the strengths and weaknesses of the proposed sequential adaptive sampling strategies with respect to traditional cross-sectional non-informative sampling as currently suggested by World Health Organisation guidelines.

## Introduction

In this paper, a novel class of sampling strategies is proposed, based on the idea of improving the quality of the sampled data by designing the sampling process on the basis of peculiar features of the surveyed population. We consider population-based surveys targeting a trait, an attribute or a condition which is at the same time difficult to detect across individuals and unevenly distributed over an area of interest, *i.e.* a study variable possibly rare and spatially clustered. Challenging issues such as budget constraints and logistics associated with the on-field operations are also taken into account, because of their importance in practice. The new sampling strategy proposed here pursues the three main aims listed below

1. Over-sampling of units that possess the characteristic of interest (positive cases, for short).
2. Cost-effectiveness, through improved control over logistics and budget’s management, both at the sample design stage and in real-time during data collection.
3. Unbiased and accurate estimation of the population mean, including unbiased variance estimation.

To meet the above aims, the use of an informative complex sampling design is proposed. In contrast to noninformative designs, as for instance traditional self-weighting equal-probability designs, a sample design is defined informative when the probability of selecting a sample (and hence the probability of a unit to be included in such sample) depends on the values of the study variable (e.g. see Cassel et al. 1977). A popular type of informative sample design is the class of Adaptive Sampling ( Thompson and Seber 1996). Our proposal is based on tailoring the data collection process by combining an adaptive approach with a sequential selection (see for instance Tillé 2006, Sect. 3.5). The adaptive component aims at purposely improving the detection of units that are positive cases. The sequential component aims at providing a flexible framework to deal with budget and logistic constraints. The two components are combined by exploiting the spatial clustering that is typical, for instance, of infectious diseases. In particular for epidemiological surveys, an obvious benefit of this approach is its increased potential to make an impact in reducing the infection burden on the surveyed population, since once detected people diagnosed as positive cases can be quarantined or/and subjected to the appropriate treatment. This paper has been originally inspired by the need of such a new sampling approach in the context of population-based surveys that measure tuberculosis (TB) prevalence at national level, which are recommended in many settings around the world and supported by World Health Organization (WHO). TB prevalence surveys will be used as a motivational example of a real application throughout the paper. However, the same perspective obviously applies to the unprecedented challenges posed by the Covid-19 planetary crisis of 2020. Indeed the pandemic has exposed worldwide the largely unmet need of providing governments, media and the general public, with truthful estimates of crucial parameters, that can not be computed upon data collected with purely medical purposes (Splendore 2020). Innovative *ad hoc* sampling strategies are strongly needed, able to monitor outbreaks and epi-curves, as well as to assess the statistical quality of data collected by health systems (see, among others, Alleva et al. 2022, Rossman et al. 2020, Franceschi et al. 2020), to analyze overall socio-economic implications, and to support official statistics (Division 2020; Radermacher 2020).

The paper is organized as follows: Sect. 2 provides the background by introducing the motivational example of prevalence surveys of TB disease. Section 3 introduces a new class of informative list-sequential adaptive sample designs for general selection of individual population units. Special attention is then paid to two important cases included in the class and based on Poisson sampling: Poisson adaptive (PoSA) design and conditional Poisson adaptive (CPoSA) design. Section 4 introduces a new class of estimators of the population mean, dubbed the Pseudo Horvitz-Thompson estimators, that are able to provide unbiased estimates under the list-sequential adaptive sampling introduced in Sect.  3. In Sect. 5 asymptotic properties of the strategies proposed in the previous sections are derived and discussed. Section 6 is devoted to illustrate the selection of primary sampling units in multi-stage designs, which is consistent with the motivational TB example. Section 7 presents empirical evidence to highlight the strengths, weaknesses and areas for improvement over traditional sampling strategies. Finally, Sect. 8 outlines our concluding remarks and future research. Proofs are gathered and fully illustrated in the Appendix.

## Background and motivation

In this section we briefly present, as an inspiring example, the population-based surveys for assessing TB prevalence at a national level, promoted by WHO and its partner agencies. Worldwide, TB is one of the top ten causes of death and the leading cause of death from a single infectious agent. The United Nations Sustainable Development Goals and the WHO’s End TB Strategy goals and targets provide the framework for national and international efforts to end the TB epidemic during the period 2016–2030. Monitoring progress against epidemiological targets is possible using evidence from national surveillance systems complemented by periodic surveys where surveillance systems are still being strengthened. Perhaps the most important of these are population-based prevalence surveys. Currently, TB prevalence surveys are being implemented according to the most recent international guidelines ( WHO 2011), where the recommended sampling design is a traditional, multi-stage, cross-sectional design. It is intended for general consumption by a wide array of practical users although, at the same time, it has limitations and inconveniences. Despite being a global public health priority (WHO 2020), statistically speaking, TB qualifies as a *rare* trait among the general population. Even in high-burden countries, national TB prevalence is generally estimated to be less than 1%. Consequently, under the currently recommended sampling design, this leads to very large sample sizes of between 50,000–100,000 people with an associated cost of USD 1–4 million. This significant investment typically leads to the estimation of a national percentage figure based on the detection of a few people diagnosed with TB among a very large sample of people without the disease. A recent example of such an outcome is given by the second national survey conducted in Vietnam 2017-2018 (Nguyen et al. 2020) where 221 (bacteriologically confirmed) TB cases have been found among the 61,763 participants in the survey (0.36% case detection). Of course, nothing is really methodologically wrong in the currently recommended traditional sampling design. However, a main stimulus behind the proposals presented in this paper is the potential for methodological improvements to optimise the investment of resources and efforts, as well as to generate additional information for TB epidemiology in the settings where the survey is implemented. The main drive for the development of a new sampling approach has been the prospect to find more people with the disease and, because TB is both infectious and mostly treatable, to isolate and cure them. Thus, it appears important to put an emphasis on over-sampling people with TB, making the survey itself a tool for reducing disease burden, generating new knowledge about TB epidemiology and informing on public health action. Equally important is the goal to gain better control over logistics. For instance, it is crucial for TB surveys that are to be implemented in the poorest settings around the world, to be able to avoid logistically difficult areas of the country, areas that might be hard to reach due to seasonal weather, flooding or even war zones. In these areas, data collection is typically compromised and the field operations budget consequently increases. In addition, we considered as a primary goal the development of a new strategy as feasible and statistically simple for use in general guidelines and field implementation. According to the inspiring TB application, our proposal can easily accomodate the selection of primary sampling units (PSU) in cluster and multistage designs. In fact, the sample design currently suggested by the WHO guidelines refers to a selection of national sub-areas as PSUs. Still, we believe that the methodology proposed here could be a useful blueprint in surveys epidemic outbreaks and other spatially clustered phenomena, attributes or conditions. With this in mind, we will first introduce our sampling strategy to select elementary units. Successively, we will illustrate how it may apply to select PSUs, consistently to our TB motivational example.

## A new class of sequential adaptive sampling designs

The goal of this section is to introduce a general class of sampling designs that is based on the idea of integrating into the List Sequential sampling (cfr. Bondesson and Thorburn 2008) an appropriate adaptive component ( Thompson 2017). Notice that the inclusion of an adaptive component considerably affects the original List Sequential scheme by Bondesson and Thorburn (2008), because the resulting sampling design becomes *informative*. For the sake of simplicity, we consider a simplified setup where the spatial setting is essentially uni-dimensional. In terms of sampling design this choice implies that the target population is pre-ordered according to a rule either natural or pre-chosen as convenient accordingly to the survey’s goals. As a result, population units would be either *close* or *distant* each other according to such an order. An example of pre-ordered population units are susceptible individuals standing in queue for service or gathering side by side in places of interest. In what follows $\ \mathcal {U} = \left\{ 1 , \, \dots \, i , \, \dots , \, N \right\}$ denotes the target population and it is also meant to reflect such an ordering. Let us denote by $\mathcal {Y}$ the character of interest, taking value $y_i$ for unit *i*. According to Sect. 2, interest is mainly in units satisfying a pre-specified condition, as for instance TB positive cases. To give a general formalisation to this fact, let *D* be a set of real numbers. Unit *i* *satisfies condition* *D* whenever $y_i \in D$. Correspondingly to unit *i*, the indicator function of the set *D*, $I_{D} (y_i )$,

$$\begin{aligned} d_i = I_{( y_{i} \in D)} = \left\{ \begin{array}{c} 1 \; {\textrm{if}} \; y_i \in D \\ 0 \; {\textrm{otherwise}} \end{array} \right. , \; i=1, \, \dots , \, N \end{aligned}$$

is considered. For instance, in terms of the TB example, each $y_i$ may be either 1 (if unit *i* is TB positive) or 0 (if unit *i* is TB negative). Thus, for binary ${\mathcal {Y}}$, we have $D = \{ 1 \}$ and $d_i = y_i$. Let $S_i$ be the sample membership indicator (SMI) of unit *i*, *i.e.* a random variable taking value $S_i=1$ if unit *i* is selected in the sample, and equal to 0 otherwise. A random sample of units is then defined as the vector of the *N* SMIs, whose value $s_1 , \, \dots , \, s_i , \, \dots , \, s_N$ will identify the selected sample. We consider the simplest choice to define the distance between units in the ordered population. *i.e.* units $i-1$ and *i*, as well as units *i* and $i+1$, are *close* for being strictly subsequent, while units $i-1$ and $i+1$ are not. We then consider the general sequential sample design in which all units $i \in \mathcal {U}$ are visited step by step along the sequence $1, \, \dots , \, N$. At step *i*, a real-time decision is made whether unit *i* is or is not selected in the sample upon the result of a Bernoulli trial. (cfr., for instance, Tillé (2006), Ch. 3). This selection scheme can be fully decribed by means of an *updating matrix*, an operative tool in the form of a ready-to-implement algorithm suitable for real-time sampling. The updating matrix lists the *N* steps of the selection process on the rows, and lists the population units on the columns in the chosen order, which also gives the visit/selection sequence. Starting from a chosen set of *initial* probabilities $\pi _i^{(0)}$, the updating matrix is given by

1 $$\begin{aligned} \begin{array}{r|cccccc} \small unit \rightarrow &{} 1 &{} 2 &{} \cdots &{} i &{} \cdots &{} N \\ step \downarrow &{} \pi _1^{(0)} &{} \pi _2^{(0)} &{} \cdots &{} \pi _i^{(0)} &{} \cdots &{} \pi _N^{(0)} \\ \hline 1 &{} S_1=s_1 &{} \pi _2^{(1)} &{} \dots &{} \pi _i^{(1)} &{} \dots &{} \pi _N^{(1)} \\ \\ 2 &{} s_1 &{} S_2=s_2 &{} \dots &{} \pi _i^{(2)} &{} \dots &{} \pi _N^{(2)} \\ \vdots &{} \dots &{} &{} \vdots &{} &{} &{} \vdots \\ i &{} s_1 &{} s_2 &{} \dots &{} S_i=s_i &{} \dots &{} \pi _N^{(i)} \\ \vdots &{} \dots &{} &{} \vdots &{} &{} &{} \vdots \\ N &{} s_1 &{} s_2 &{} \dots &{} s_i &{} \dots &{} S_n=s_N \\ \\ \end{array} \end{aligned}$$

It is important to notice that unit *i* may be selected/not selected only when visited at the *i*-th step of the sampling algorithm, i.e at the *i*-th row of the updating matrix (1). Therefore, at each step of the selection sequence, the matrix entries can be in one of the three following states.

1. Before visiting unit *i* (i.e. until step $i-1$, lower triangle), the sample membership of unit *i* is the r.v. $S_i$.
2. At step *i*, unit *i* is visited and its selection/not selection attained, *i.e.* the sample membership indicator takes on its realisation $S_i=s_i$.
3. After (*i.e.* from step $i+1$ on) the actual sample membership $s_i$ is recorded for unit *i* with no more randomness.

At the end of the selection process, the last row of the updating matrix shows the selected sample. Moreover, upon recording $S_i=s_i$ along the diagonal of the updating matrix, the selection probabilities are updated for all subsequent to-be-visited units, $j=i+1 , \, \dots , \, N$ (upper triangle) according to a chosen *updating rule*. In our proposal for an informative sequential sampling we propose to use an *adaptive* updating rule. According to our goal to over-sampling units satisfying condition *D*, and given that in many cases of interest, such as in the TB example, they can be expected to be close each other along the sequence of population units (in the order they are visited), the decision to include/not include unit *i* in the sample is made depending on the result of the previous step. This leads to the following adaptive updating rule

2 $$\begin{aligned} \pi _j^{(i)}= \left\{ \begin{array}{ll} 1 &{} \text {if} \quad j=i+1 \text { and } S_i d_i=1 \\ \pi _j^{(i-1)} - \left( S_i - \pi _i^{(i-1)} \right) w^{(i)}_{j-i} &{} \text {otherwise} \end{array} \right. \end{aligned}$$

Quantities $w^{(i)}_{j-i}$ are the updating weights, chosen in order to satisfy the constraints $0 < \pi _j^{(i)} \le 1$. Hence, Eqs. 1 and 2 define an entire class of sequential adaptive sampling algorithms according to different choices for the updating weights $w^{(i)}_{j-i}$. In the sequel, we will always assume that $w^{(i)}_{j-i}$ depends on $S_1$, $\dots$, $S_{i-1}$, as well as on $y_j$s corresponding to $S_j =1$, namely on the pairs $(( S_j , \, S_j y_j); \; j=1, \, \dots , \, i)$. More formally, if $\mathcal {F}_i = \sigma ( S_1 , \, \dots , \, S_i )$ is the sub-$\sigma$-field generated by $S_1, \, \dots , \, S_i$, the weights $w^{(i)}_{j-i}$ are assumed to be measurable w.r.t. $\mathcal {F}_{i-1}$. Furthermore, from Eq. (2) it follows that

3 $$\begin{aligned} P( S_i = s \vert \mathcal {F}_{i-1} ) = \left( \pi ^{(i-1)}_i \right) ^s \left( 1- \pi ^{(i-1)}_i \right) ^{1-s} , \;\; s \in \{ 0, \, 1\} . \end{aligned}$$

In the following subsections we propose two special cases of the informative sequential adaptive design above that are of practical interest, and we develop the associated probabilistic input required for unbiased estimation.

### Poisson sequential adaptive (PoSA) sampling design

Poisson Sequential Adaptive (PoSA, for short) sampling design is included into the general scheme introduced above by taking null weights $w^{(i)}_{j-i}$, which leads to the following PoSA updating rule:

4 $$\begin{aligned} \pi _j^{(i)}=\left\{ \begin{array}{cl} d_i S_i + \pi _{i+1}^{(0)}\left( 1-d_i S_i\right) &{} \text {if} \quad j=i+1\\ \\ \pi _j^{(0)} &{} \text {if}\quad j > i+1 \end{array} \right. \end{aligned}$$

According to (4) if, at step *i*, unit *i* is selected $(S_i=1)$ *and* it results in a positive case ($d_i=1$), then the selection probability of the close unit $i+1$ is updated to 1, so that it is certainly included in the sample. Otherwise, the selection probability of the close unit $i+1$ is left unaltered to its initial value, similarly to all the remaining selection probabilities of units $j > i+1.$ In this way the updating is limited because it actually affects only pairs of strictly subsequent units. A first important feature of PoSA is that it possesses a Markov-type property

5 $$\begin{aligned} P\left( S_i= s \big | \mathcal {F}_{i-1}\right) = P \left( \left. S_i= s \right| S_{i-1} \right) = \left( \pi _i^{(i-1)}\right) ^s \left( 1- \pi _i^{(i-1)} \right) ^{1-s} \;\;\; s \in \{ 0, \, 1 \} . \end{aligned}$$

According to (5), unit *i* is selected at step *i* with conditional probability $\pi _{i}^{(i-1)}$, which is known at the previous step, located at the previous row, same column in the updating matrix (1).

In the subsequent Proposition 1, first and second order (unconditional) inclusion probabilities for PoSA design are computed

#### Proposition 1

For PoSA design, first and second order inclusion probabilities are equal to

6 $$\begin{aligned} \pi _i= & {} E \left[ S_i\right] = \pi _{i}^{(0)} + \sum _{j=1}^{i-1} \pi _j^{(0)} \prod _{h=j+1}^{i} \left( 1-\pi _h^{(0)}\right) d_{h-1} \end{aligned}$$

7 $$\begin{aligned} \pi _{i , i+k}= & {} E \left[ S_i S_{i+k}\right] = \pi _i \left\{ \pi ^{(0)}_{i+k} + \sum _{j=i+1}^{i+k-1} \pi _j^{(0)} \prod _{h=j}^{i+k-1} \left( 1- \pi ^{(0)}_{h+1}\right) d_h \right. \nonumber \\{} & {} \left. + \prod _{h=i}^{i+k-1} \left( 1- \pi ^{(0)}_{h+1} \right) d_h \right\} . \;\; \end{aligned}$$

Note that the inclusion probabilities ( 6 ), (7) only depend on the initial probabilities $\pi ^{(0)}_i$s and on $d_i$s. In this sense, PoSA design does not depend on the order of units. However, the conditional probabilities $P\left( S_i= s \big | \mathcal {F}_{i-1}\right)$ do depend on the order of units.

Of course, unconditional inclusion probabilities cannot be computed in practice, because they also depend of $d_i$s associated to unobserved population units. Furthermore, from Proposition 1 it is easy to verify that

8 $$\begin{aligned} \pi _{i,i+k} - \pi _i \pi _{i+k} = \pi _i (1- \pi _i ) (1- \pi ^{(0)}_{i+1} ) \cdots (1- \pi ^{(0)}_{i+k} ) d_{i} \cdots d_{i+k-1} . \end{aligned}$$

Equation ( 8 ) shows that $\pi _{i,i+k} = \pi _i \pi _{i+k}$ if at least one among $d_i , \, \dots , \, d_{i+k-1}$ is equal to 0. Since $S_i$s are Bernoulli r.v.s, this implies that $S_i$ and $S_{i+k}$ are independent if at least one among $d_i , \, \dots , \, d_{i+k-1}$ is equal to 0. In addition, $S_i$, $S_{i+1}$, $\dots$, $S_{i+k}$ are jointly independent when $d_i , \, \dots , \, d_{i+k-1}$ are all equal to 0. To clarify further the structure of inclusion probabilities for PoSA design, let us first observe that the *N* population units can be partitioned into consecutive blocks

9 $$\begin{aligned} B^0_1 , \, B^1_1 , \, B^0_2 , \, B^1_2 , \, \dots , \, B^0_K , \, B^1_K \end{aligned}$$

where each block $B^0_k$ is composed by consecutive units with $d_i = 0$, and each block $B^1_k$ is composed by consecutive units with $d_i =1$, $k=1, \, \dots , \, K$. Note that $B^0_1$ and/or $B^1_K$ may be empty. To simplify the notation, from now on we will denote by $i_{k,j}$ the first unit of block $B^j_{k}$, $j=0, \, 1$. From ( 6 ) it follows

10 $$\begin{aligned} \pi _i = \left\{ \begin{array}{ll} \pi _i^{(0)} &{} \text {if} \quad i \in B^{0}_{1} \\ \pi ^{(0)}_i &{} \text {if} \quad i \in ( B^{0}_k \setminus \{ i_{k,0} \} ) \cup \{ i_{k+1, 1} \} , \;\; k >1 \\ \pi ^{(0)}_i + \sum _{j=i_{k,1}}^{i-1} \pi ^{(0)}_j \prod _{h=j+1}^{i} (1- \pi ^{(0)}_h ) &{} \text {if} \quad i \in ( B^{1}_k \setminus \{ i_{k,1} \} ) \cup \{ i_{k+1, 0} \} , \;\; k \ge 1 \\ \end{array} \right. \end{aligned}$$

Let $\varvec{s}$ denote the selected sample, namely the set of *i* such that the last $N^{th}$ row of the updating matrix (1) shows a value $s_i$ equal to 1. PoSA sampling results in a sample of random size $n_{\varvec{s}}$, given by

11 $$\begin{aligned} n_{\varvec{s}} = \sum _{i=1}^N S_i . \end{aligned}$$

The expected sample size is then equal to

12 $$\begin{aligned} E \left[ n_{\varvec{s}} \right] = \sum _{i=1}^N \ \pi _i^{(0)} + \sum _{i=2}^N \left( 1-\pi _i^{(0)} \right) d_{i-1}E \left( S_{i-1} \right) = \sum _{i=1}^N \ \pi _i^{(0)} + \sum _{i=2}^N \left( 1-\pi _i^{(0)} \right) d_{i-1} \pi _{i-1} . \end{aligned}$$

PoSA expected sample size depends on unknown population quantities, though it is lower-bounded by $\sum _{i=1}^N \pi _i^{(0)}$. This will be further discussed in the next section.

### Controlling sample size: conditional poisson sequential adaptive (CPoSA) design

Although PoSA sampling design is of practical interest for its simplicity, its random sample size $n_{\varvec{s}}$ can limit its applicability. In principle, the random sample size is a characteristic of adaptive sampling and randomness around the PoSA sample size is a natural by-product of its effectiveness in over-sampling positive cases. However it is often regarded as a practical issue and, as a consequence of PoSA simplicity, it possibly leads to extreme results. In particular, it could give unnecessarily large samples even if no new positive cases are detected; at the same time, it may threaten estimates accuracy when leading to overly small samples. With the purpose to remove such extremes and to enhance control over sample size, we now introduce a conditional version of PoSA, dubbed CPoSA. Meanwhile, we do not want to compromise on desirable features of PoSA, namely the over-sampling of positive cases, its technical simplicity and readiness to implement. We therefore target two key points: first of all, we want to avoid unacceptable small samples. To this end, a *minimum* sample size should be established so that the selection process is not allowed to stop before this minimum has been reached. In the second place, additional selections would be still allowed provided that they are additional positive cases. Let $n_{min}$ be the pre-fixed minimum sample size. The set of initial probabilities has to be chosen such that $\sum _{i=1}^N \pi _i^{(0)}=n_{min}$. Then, CPoSA selection has to be similar to PoSA in that, at the *i*-th step, $(i \ge 2)$ unit *i* is selected with the probability updated at the previous step $\pi _i^{(i-1)}$. However, a modification in the CPoSA updating rule makes the design both adaptive and dependent on the number *i* of units already visited, to enforce the over-sampling of positive cases while securing at least sample size $n_{min}$. A simple way to accomplish both requirements above is to include a weighting factor $1/(N-i)$ into the PoSA adaptive updating rule (4). Moreover, if at step *i*, unit *i* is certainly included in the sample as a result of the adaptive mechanism, *i.e.* because of its closeness to a selected positive case, it should count as an additional selection and shall not impact changes on the selection probabilities of non-strictly subsequent units. This gives the following CPoSA updating rule, that applies, at step $i \ge 2$, to all units $j \ge i+1$

13 $$\begin{aligned} \pi _j^{(i)}= \left\{ \begin{array}{ll} 1 &{} \text {if} \quad j=i+1 \text { and } S_i d_i=1 \\ \pi _j^{(i)}= \max \left( 0, \, \min \left( \pi _j^{(i-1)} - \frac{S_i - \pi _i^{(i-1)}}{N-i} , \, 1 \right) \right) &{} \text {if} \quad j >i+1 \end{array} \right. \end{aligned}$$

A main difference of CPoSA with respect to PoSA is that in the $i^{th}$ row of the updating matrix, all units $j > i+1$ undergo an actual updating of selection probabilities. In particular

14 $$\begin{aligned} \begin{array}{ll} \pi _{i+1}^{(i)} = 1 \quad &{} \text { if} \quad S_i d_i=1 \\ \\ \pi _j^{(i)} \le \pi _j^{(i-1)} &{} \text {if} \quad j \ge i + 2, \ S_i = 1, \ d_i=0, \, 1 \\ \\ \pi _j^{(i)} \ge \pi _j^{(i-1)} &{} \text {if} \quad j \ge i + 2, \ S_i = 0, \ d_i=0, \, 1 \\ \end{array} \end{aligned}$$

Therefore, updated selection probabilities would increase in case of non-selection of visited units in order to pursue the stated minimum sample size. At the same time, updated selection probabilities would tend to 0 in case of consecutive selections of negative cases beyond the $n_{min}$ bound. In other words, CPoSA sampling, despite providing a final sample of random size to foster its ability to over-sample positive cases, aims at a sample of at least size $n_{min}$ while avoiding to inflate the final sample with additional negative cases. This remark clarifies how CPoSA can increase control, with respect to PoSA, upon both excessively small *and* large effective sample sizes. To gain further control on large sample behaviour, we will also consider the following slight modification of CPoSA updating rule (13)

15 $$\begin{aligned} \pi _j^{(i)}= \left\{ \begin{array}{ll} 1 &{} \text {if} \quad j=i+1 \text { and } S_i d_i=1 \\ \pi _j^{(i)}= \max \left( \delta _N , \, \min \left( \pi _j^{(i-1)} - \frac{S_i - \pi _i^{(i-1)}}{N-i} , \, 1 \right) \right) &{} \text {if} \quad j >i+1 \\ \end{array} \right. \end{aligned}$$

with $\delta _N >0$ for each positive *N* that could either decrease to zero or to a positive $\delta$ as *N* increases.

The behaviour of CPoSA, mainly in terms of control on the final sample size, will be further illustrated in Sect. 7 by simulation results.

## Unbiased estimation of the population mean under informative sequential adaptive sampling: the Pseudo-HT estimator

In the present section we focus on the population mean $\overline{y}_N = \sum _{i=1}^N y_i / N$ as the parameter of interest. The case of a proportion, corresponding to the motivating example of TB prevalence surveys (prevalence is defined as the proportion of positive cases in a given country at a give point in time), is a special case for binary $y_i$s.

In order to construct an unbiased estimator of $\overline{y}_N$, consider the conditional inclusion probabilities $\pi _i^{(i-1)} = E \left[ S_i \vert \mathcal {F}_{i-1} \right]$, and define the r.v.s

16 $$\begin{aligned} T_{i} = \frac{S_i}{\pi _i^{(i-1)}} -1 , \;\; i=1, \, \dots , \, N . \end{aligned}$$

The probability distribution of $T_i$, conditionally on $\mathcal {F}_{i-1}$, is known, because it only depends on observed quantities, namely $(S_1 , \, S_1 d_1 )$, $\dots$, $( S_{i-1} , \, S_{i-1} d_{i-1} )$. The most important property of r.v.s $T_i$s is reported in Proposition 2.

### Proposition 2

Let $1 \le n \le N$, and consider *n* indices $1 \le i_{1}< i_{2}< \cdots < i_{n} \le N$. Then, the relationship

17 $$\begin{aligned} E \left[ T_{i_{1}} T_{i_{2}} \cdots T_{i_{n}} \right] =0 \end{aligned}$$

holds.

As a by-product of Proposition 2 it readily follows that the r.v.s $T_i$s are pair-wise uncorrelated:

18 $$\begin{aligned} C \left( T_i , \, T_j \right) =0 \;\; i \ne j =1 , \, \dots , \, N \end{aligned}$$

$C( \cdot , \cdot )$ denoting the covariance operator.

Proposition 2 essentially shows that $(( T_1 , \, \dots , \, T_N ) ; \; N \ge 1 )$ is a martingale difference array; cfr. Hall and Heyde (1980).

As an estimator of $\overline{y}_N$ we consider here the Pseudo Horvitz-Thompson (Pseudo-HT, for short) estimator:

19 $$\begin{aligned} \widehat{\bar{Y}}_{PHT} = \frac{1}{N} \sum _{i=1}^{N} \frac{S_i}{\pi _i^{(i-1)}} y_i . \end{aligned}$$

Expectation and variance of $\widehat{\bar{Y}}_{PHT}$ are summarized in Proposition 3, which is readily obtained from Proposition  2 and Eq. (18).

### Proposition 3

The expectation and the variance of $\widehat{{\bar{Y}}}_{PHT}$ are equal to

20 $$\begin{aligned} E \left[ \widehat{\bar{Y}}_{PHT} \right]= & {} \overline{y}_N \end{aligned}$$

21 $$\begin{aligned} V \left( \widehat{\bar{Y}}_{PHT} \right)= & {} \frac{1}{N^2} \sum _{i=1}^{N} E \left[ \frac{1}{\pi _i^{(i-1)}} -1 \right] y_i^2 \end{aligned}$$

respectively.

From Eq. ( 21 ) it is also easy to see that an unbiased estimator of the variance of $\widehat{\bar{Y}}_{PHT}$ is

22 $$\begin{aligned} \widehat{V}_{PHT} = \frac{1}{N^2} \sum _{i=1}^{N} \frac{S_{i}}{\pi _i^{(i-1)}} \left( \frac{1}{\pi _i^{(i-1)}} -1 \right) y_i^2 . \end{aligned}$$

### PoSA Pseudo-HT estimation

In this section Pseudo-HT estimation is applied under PoSA sampling design proposed in Sect. 3.1. The relations with the plain (non-adaptive, non-informative) Poisson design will be studied and gain in efficiency of PoSA versus Poisson sampling will be illustrated.

Under PoSA design, since $\pi ^{(i-1)}_i = \pi ^{(0)}_{i} + d_{i-1} S_{i-1} ( 1 - \pi ^{(0)}_{i} )$ is either equal to $\pi _i^{(0)}$ or to 1, it follows that

23 $$\begin{aligned} \frac{1}{\pi ^{(i-1)}_{i}} = d_{i-1} S_{i-1} \left( 1 - \frac{1}{\pi ^{(0)}_{i}} \right) + \frac{1}{\pi ^{(0)}_{i}} \end{aligned}$$

and hence the estimator $\widehat{\bar{Y}}_{PHT}$ can be written as

24 $$\begin{aligned} \widehat{\bar{Y}}_{PoSA}= & {} \frac{1}{N} \sum _{i=1}^{N} \left\{ d_{i-1} S_{i-1} \left( 1 - \frac{1}{\pi ^{(0)}_{i}} \right) + \frac{1}{\pi ^{(0)}_{i}} \right\} S_i y_i \nonumber \\= & {} \frac{1}{N} \sum _{i=1}^{N} \frac{S_{i}}{\pi ^{(0)}_{i}} y_i + \frac{1}{N} \sum _{i=1}^{N} \left( 1- \frac{1}{\pi ^{(0)}_{i}} \right) d_{i-1} S_{i-1} S_i y_i \end{aligned}$$

with $d_0 =0$. The term $N^{-1} \sum S_i y_i / \pi ^{(0)}_{i}$ is essentially the Horvitz-Thompson estimator of $\overline{y}_N$ in case of (non-informative) Poisson design with inclusion probabilities $\pi ^{(0)}_i$s. The second term in (24 ) is an additional term due to the adaptive updating rule and the use of conditional inclusion probabilities. As far as the variance of (24 ) is concerned, from (21 ) and (23) it follows

$$\begin{aligned} V \left( \widehat{\bar{Y}}_{PoSA} \right)= & {} \frac{1}{N^{2}} \sum _{i=1}^{N} E \left[ \frac{1}{\pi ^{(0)}_{i}} -1 + d_{i-1} S_{i-1} \left( 1 - \frac{1}{\pi ^{(0)}_{i}} \right) \right] y_i^2 \\= & {} \frac{1}{N^{2}} \sum _{i=1}^{N} \left( \frac{1}{\pi ^{(0)}_{i}} -1 \right) y^2_i - \frac{1}{N^{2}} \sum _{i=1}^{N} \left( \frac{1}{\pi ^{(0)}_{i}} -1 \right) d_{i-1} E[ S_{i-1} ] y_i^2 \\= & {} \frac{1}{N^{2}} \sum _{i=1}^{N} \left( \frac{1}{\pi ^{(0)}_{i}} -1 \right) y^2_i - \frac{1}{N^{2}} \sum _{i=1}^{N} \left( \frac{1}{\pi ^{(0)}_{i}} -1 \right) d_{i-1} \pi _{i-1} y_i^2 . \end{aligned}$$

The term

$$\begin{aligned} \frac{1}{N^{2}} \sum _{i=1}^{N} \left( \frac{1}{\pi ^{(0)}_{i}} -1 \right) y_i^2 \end{aligned}$$

is the variance of the Horvitz-Thompson estimator if Poisson design with inclusion probabilities $\pi ^{(0)}_i$s were to be used. The term

$$\begin{aligned} \frac{1}{N^{2}} \sum _{i=1}^{N} \left( \frac{1}{\pi ^{(0)}_{i}} -1 \right) d_{i-1} \pi _{i-1} y_i^2 \end{aligned}$$

represents the gain in efficiency (reduction of variance) under PoSA design.

Similar considerations, with more complicated formulas, can be done under CPoSA design illustrated in Sect. 3.2.

## Asymptotic properties under informative sequential adaptive sampling

### Generalities and assumptions

The present section is devoted to study asymptotic properties of the estimator $\widehat{\bar{Y}}_{PHT}$ when the population size *N* increases. The assumptions on which asymptotic normality for PoSA design rests are listed below, where the symbol

25 $$\begin{aligned} \sigma _N^2= & {} \sum _{i=1}^{N} E \left[ \frac{1}{\pi ^{(i-1)}_{i}} -1 \right] y^2_i \end{aligned}$$

is used.

1. $\delta \le \pi ^{(0)}_i \le 1- \delta$ for some $\delta >0$, and for each $N \ge 1$ and $i=1, \, \dots , \, N$.
2. The limits
  $$\begin{aligned} \mu _{y} = \lim _{N \rightarrow \infty } \frac{1}{N} \sum _{i=1}^{N} y_i , \;\; \mu _{2y} = \lim _{N \rightarrow \infty } \frac{1}{N} \sum _{i=1}^{N} y_i^2 \end{aligned}$$
  exist, with $$\mu _{2y} > 0$$.
3. Non-null asymptotic variance:
  26 $$\begin{aligned} \liminf _{N \rightarrow \infty } N^{-1} \sigma _N^2 >0 . \end{aligned}$$
4. The values $y_i$s are uniformly bounded by a constant *M*: $\vert y_i \vert \le M$ for each $i=1, \, \dots , \, N$ and $N \ge 1$.
5. $\vert B^{1}_{k} \vert / \sqrt{N} = o(1)$ uniformly in *k*, as $N \rightarrow \infty$.

#### Remark 1

Assumptions A1, A2 are quite standard for asymptotics in survey sampling; cfr., for instance, Francisco and Fuller (1991) and Isaki and Fuller (1982). Assumption A3 is a slightly weaker than Condition 3 (which involves, in its turn, a superpopulation model) in Francisco and Fuller (1991).

In the subsequent sections consistency and asymptotic normality of the Pseudo-HT estimator are established. The attack line is fairly simple. Asymptotic properties are first studied for PoSA design, and then extended to more general (informative) sequential adaptive designs.

#### Remark 2

Under PoSA design, from the inequality, $\pi ^{(i-1)}_i \ge \pi ^{(0)}_i \ge \delta$ we get

$$\begin{aligned} \frac{1}{N} \sigma ^2_N \le \frac{1}{N} \sum _{i=1}^{N} \left( \frac{1}{\delta } -1 \right) y^2_i \end{aligned}$$

and hence, using Assumption A2, it is seen that

$$\begin{aligned} \limsup _{N \rightarrow \infty } \frac{1}{N} \sigma ^2_N \le \frac{1- \delta }{\delta } \mu _{2y} < \infty . \end{aligned}$$

As a consequence of A3, there exist two positive constants *c*, *C* such that

27 $$\begin{aligned} c N \le \sigma ^2_N \le C N \end{aligned}$$

for all *N*s large enough.

#### Remark 3

Assumption A5 is fulfilled, for instance, when values $y_i$s are generated according to a superpopulation model where the r.v.s $Y_i$s are *i.i.d.*. In this case, the values $d_i$s are realizations of *i.i.d.* Bernoulli r.v.s $D_i = I_{D} ( Y_i )$. Clearly, the blocks ( 9 ) correspond to (maximal) runs of 0s and 1s, respectively. Let $\vert B^1_k \vert$ be the cardinality of block $B^1_k$, so that $\max _{1 \le k \le K} \vert B^1_k \vert$ is the length of the longest run of 1s in the sequence $d_1 , \, \dots , \, d_N$. Define next $p= \mathbb {P} ( D_i =1 )$, and assume $p>0$. From the Erdös and Rényi (1970) law of large numbers we get

$$\begin{aligned} \max _{1 \le k \le K} \vert B^1_k \vert / \log _{1/p} N \rightarrow 1 \end{aligned}$$

with $\mathbb {P}$-probability 1. Furthermore, an elementary computation shows that the total number of runs has expected value $1 + 2 (N-1) p (1-p)$, and that its variance is smaller than *constN* for an appropriate $const >0$, so that $K /N = O (1)$ with $\mathbb {P}$-probability 1.

### Consistency and asymptotic normality: PoSA design

The consistency of $\widehat{\bar{Y}}_{PoSA}$ is derived under a condition slightly weaker than A1.

#### Proposition 4

Suppose that $\pi ^{(0)}_i \ge \delta _N$, for all $i=1, \, \dots , \, N$, with $N \delta _N \rightarrow \infty$ as $N \rightarrow \infty$. Then

28 $$\begin{aligned} \widehat{\bar{Y}}_{PoSA} - \overline{y}_N {\mathop {\rightarrow }\limits ^{p}} 0 \;\; {\textrm{as}} \; N \rightarrow \infty \end{aligned}$$

where ${\mathop {\rightarrow }\limits ^{p}}$ denotes convergence in probability.

Notice that condition $\pi ^{(0)}_i > \delta _N$ implies that the unconditional inclusion probabilities $\pi _i = E [ \pi ^{(i-1)}_i ]$ are all larger than $\delta _N$. Hence the expected sample size satisfies the inequality:

$$\begin{aligned} E \left[ n_{\varvec{s}} \right] = \sum _{i=1}^{N} \pi _i \ge N \delta _N . \end{aligned}$$

This means that the expected sample size has to tend to infinity as *N* does. On the other hand, the expected sampling fraction $E \left[ n_{\varvec{s}} \right] / N$ is larger than $\delta _N$, and hence it can either tend to 0 or to a positive number as *N* increases.

Let us now move to asymptotic normality under PoSA design. To simplify the notation, define

29 $$\begin{aligned} X_{Ni} = \frac{\sqrt{N}}{\sigma _{N}} \left( \frac{S_{i}}{\pi _{i}^{(i-1)}} -1 \right) y_i , \;\; i=1, \, \dots , \, N . \end{aligned}$$

Clearly, $( ( X_{Ni} ; \; i=1, \, \dots , \, N) ; \; N \ge 1)$ is a martingale difference array.

#### Lemma 1

Under assumptions A1-A5 and PoSA design:

30 $$\begin{aligned} \frac{1}{N} \sum _{i=1}^{N} E [ X_{Ni}^2 \vert \mathcal {F}_{i-1} ] {\mathop {\rightarrow }\limits ^{p}} 1 \;\; {\textrm{as}} \; N \rightarrow \infty . \end{aligned}$$

#### Lemma 2

Under assumptions A1-A5 and PoSA design, there exists a positive constant *R* for which

31 $$\begin{aligned} E[ X_{Ni}^4 ] \le R \;\; \forall i=1, \, \dots , \, N \; {\textrm{and}} \; \forall N \ge 1 . \end{aligned}$$

#### Proposition 5

Suppose that Assumptions A1-A5 are fulfilled. Under PoSA design, the following three statements hold.

32 $$\begin{aligned}&\,&\frac{\widehat{\bar{Y}}_{PoSA} - \overline{y}_N}{\sqrt{V (\widehat{\bar{Y}}_{PoSA})}} {\mathop {\rightarrow }\limits ^{d}} N(0, \, 1 ) \;\; {\textrm{as}} \; N \rightarrow \infty ; \end{aligned}$$

33 $$\begin{aligned}&\,&\frac{\widehat{V}_{PHT}}{V (\widehat{\bar{Y}}_{PoSA})} {\mathop {\rightarrow }\limits ^{p}} 1 \;\; {\textrm{as}} \; N \rightarrow \infty ; \end{aligned}$$

34 $$\begin{aligned}&\,&\frac{\widehat{\bar{Y}}_{PoSA} - \overline{y}_N}{\sqrt{\widehat{V}_{PHT}}} {\mathop {\rightarrow }\limits ^{d}} N(0, \, 1 ) \;\; {\textrm{as}} \; N \rightarrow \infty , \end{aligned}$$

$N(0, \, 1)$ denoting the Standard Normal distribution, ${\mathop {\rightarrow }\limits ^{d}}$ denoting convergence in distribution, and ${\mathop {\rightarrow }\limits ^{p}}$ convergence in probability.

#### Remark 4

As remarked by a referee, Proposition 5 may be proved differently. Since

$$\begin{aligned} \left( \frac{S_i}{\pi _{i}^{(i-1)}} \right) _{i \in B_{1}^{1} \cup \{ i_{2,0} \} } , \, \dots , \, \left( \frac{S_i}{\pi _{i}^{(i-1)}} \right) _{i \in B_{1}^{K-1} \cup \{ i_{K,0} \} } , \, \left( \frac{S_i}{\pi _{i}^{(i-1)}} \right) _{i \in B_{1}^{K}} \end{aligned}$$

and

$$\begin{aligned} \frac{S_i}{\pi _{i}^{i-1)}} , \; i \in B_1^0 \cup \left( \bigcup _{k=2}^{K} B^0_k \setminus \{ i_{k,0} \} \right) \end{aligned}$$

are two independent sequences of independent r.v.s, the Central Limit Theorem for triangular arrays of independent r.v.s could be used.

### Consistency and asymptotic normality: general sequential adaptive designs

This section deals with consistency and asymptotic normality of $\widehat{\bar{Y}}_{PHT}$ under rather general sequential adaptive designs, including CPoSA.

In Lemma 3, which is used in all subsequent developments, the relationships between a general sequential adaptive design and PoSA design are studied in terms of difference of conditional inclusion probabilities.

#### Lemma 3

Consider a general sequential adaptive design with updating rule (2), with initial probabilities $\pi ^{(0)}_i$ and conditional inclusion probabilities $\pi ^{(i)}_j$. Let further $\pi ^{(i)}_{j,PoSA}$ be the conditional inclusion probabilities of a PoSA design with the same initial probabilities $\pi ^{(0)}_i$. Then, the following relationship holds:

35 $$\begin{aligned} \pi ^{(i)}_{i+1} = S_i d_i + (1- S_i d_i ) \pi ^{(0)}_{i+1} - C_{N,i} = \pi ^{(i)}_{i+1,PoSA} - C_{N,i} \end{aligned}$$

where

36 $$\begin{aligned} c_{ik}= & {} (1- S_i d_i ) \left( S_{i-k+1} - \pi ^{(i-k)}_{i-k+1} \right) w^{(i-k+1)}_{k} , \;\; k \ge 1 \end{aligned}$$

37 $$\begin{aligned} C_{N,i}= & {} c_{i1} + \cdots + c_{ii} . \end{aligned}$$

Moreover, the inequality

38 $$\begin{aligned} E \left[ \vert C_i \vert \right] \le A_{N,i} , \;\; i =1, \, \dots , \, N \end{aligned}$$

holds, with

39 $$\begin{aligned} A_{N,i} = \sqrt{\sum _{k=1}^{i} E \left[ \left( w^{(i-k+1)}_{k} \right) ^2 \right] } . \end{aligned}$$

In order to get consistency the main restrictions we consider are in Assumptions A6, A7 below.

A6 Consider the weights of the updating rule (2 ), and let $A_{N,i}$ be defined as in (39 ). Then, $\sum _{i=1}^{N} A_{N,i-1} = o(N)$ as $N \rightarrow \infty$.
A7 $\pi ^{(i-1)}_i \ge \delta _N$ for every $i=1, \, \dots , \, N$ and $N \ge 1$, with $N \delta _N^2 \rightarrow \infty$ as $N \rightarrow \infty$.

#### Example 1

Consider the CPoSA design, with $w^{(i)}_k = 1 / (N-i)$. The term $A_{N,i}^2$ is easily computed by taking into account that

$$\begin{aligned} A_{N,i}^2= & {} \sum _{k=1}^{i} \frac{1}{(N-(i-k+1))^{2}} = \sum _{j=1}^{i} \frac{1}{(N-j)^2} \\\le & {} \frac{1}{N^{2}} \sum _{j=0}^{i} \frac{1}{(1-j/N)^2} \le \frac{1}{N} \int _{0}^{i/N} \frac{1}{(1-x)^{2}} \, dx \\= & {} \frac{1}{N} \left( \frac{1}{1- i/N} -1 \right) = \frac{1}{N} \frac{i}{N-i} \end{aligned}$$

from which we get

40 $$\begin{aligned} A_{N,i} \le \frac{1}{\sqrt{N}} \sqrt{\frac{i}{N-i}}. \end{aligned}$$

Next, from

$$\begin{aligned} \frac{1}{N} \sum _{i=1}^{N} A_{N, i-1}= & {} \frac{1}{N^{3/2}} \sum _{i=1}^{N} \sqrt{\frac{i}{N-i+1}} = \frac{1}{N^{3/2}} \sum _{i=1}^{N} \left( \frac{i}{N} \right) ^{1/2} \left( 1- \frac{i-1}{N} \right) ^{-1/2} \\\le & {} \frac{1}{\sqrt{N}} B( 3/2 , \, 1/2 ) , \end{aligned}$$

where $B(u, \, v)$ is the Beta function of arguments *u*, *v*, it is seen that Assumption A6 is satisfied.

#### Proposition 6

Suppose that Assumptions A1-A7 are fulfilled and that $\pi ^{(0)}_i \ge \delta _N$, for all $i=1, \, \dots , \, N$, with $N \delta _N \rightarrow \infty$ as $N \rightarrow \infty$. Then:

41 $$\begin{aligned} \widehat{\bar{Y}}_{PHT} - \overline{y}_N {\mathop {\rightarrow }\limits ^{p}} 0 \;\; {\textrm{as}} \; N \rightarrow \infty . \end{aligned}$$

To get asymptotic normality we need a condition stronger than A7, which is reported below.

A7$$^{\prime }$$
$\pi ^{(i-1)}_i \ge \delta$ for every $i=1, \, \dots , \, N$ and $N \ge 1$.

#### Lemma 4

Under Assumptions A1-A6 and A7$^{\prime }$ we have

42 $$\begin{aligned} \frac{1}{N} \sum _{i=1}^{N} \left( \frac{1}{\pi ^{(i-1)}_{i}} - \frac{1}{\pi ^{(i-1)}_{i,PoSA}} \right) y_i^2 {\mathop {\rightarrow }\limits ^{p}} 0 \;\; {\textrm{as}} \; N \rightarrow \infty . \end{aligned}$$

Consider $\sigma _N^2$ and $X_{Ni}$ as defined in (25) and (29), respectively.

#### Lemma 5

Under assumptions A1-A6 and A7$^{\prime }$, the following two results hold:

43 $$\begin{aligned} \frac{1}{N} \sum _{i}^{N} E \left[ X_{Ni}^2 \vert \mathcal {F}_{i-1} \right] {\mathop {\rightarrow }\limits ^{p}} 1 \;\; {\textrm{as}} \; N \rightarrow \infty . \end{aligned}$$

44 $$\begin{aligned} E[ X_{Ni}^4 ] \le R \;\; \forall i=1, \, \dots , \, N \; {\textrm{and}} \; \forall N \ge 1 . \end{aligned}$$

#### Proposition 7

Suppose that Assumptions A1-A6 and A7$^{\prime }$ are fulfilled. Then, the following three statements hold.

45 $$\begin{aligned}&\,&\frac{\widehat{\bar{Y}}_{PHT} - \overline{y}_N}{\sqrt{V (\widehat{\bar{Y}}_{PHT})}} {\mathop {\rightarrow }\limits ^{d}} N(0, \, 1 ) \;\; {\textrm{as}} \; N \rightarrow \infty ; \end{aligned}$$

46 $$\begin{aligned}&\,&\frac{\widehat{V}_{PHT}}{V (\widehat{\bar{Y}}_{PHT})} {\mathop {\rightarrow }\limits ^{p}} 1 \;\; {\textrm{as}} \; N \rightarrow \infty ; \end{aligned}$$

47 $$\begin{aligned}&\,&\frac{\widehat{\bar{Y}}_{PHT} - \overline{y}_N}{\sqrt{\widehat{V}_{PHT}}} {\mathop {\rightarrow }\limits ^{d}} N(0, \, 1 ) \;\; {\textrm{as}} \; N \rightarrow \infty . \end{aligned}$$

#### Remark 5

As it appears from Example 1, CPoSA design satisfies Assumption A6. In addition, the modified CPoSA updating rule ( 15 ) with $\delta _N = \delta >0$ also satisfies Assumption A7$^{\prime }$.

#### Remark 6

Propositions 6, 7 also hold, with minor adaptations in the original non-informative setting by Bondesson and Thorburn (2008). Thus, they also provide a way to prove consistency and asymptotic normality of the pseudo-HT estimator in the class of non-informative designs by Bondesson and Thorburn (2008). Since this point is outside of the goals of the present paper, we do not pursue into that direction.

#### Remark 7

If $y_i$ values, as well as the initial inclusion probabilities $\pi _i^{(0)}$ are allowed to depend on *N*, under conditions A1-A5, all results of the present section still hold true. This will be used in Sect. 6 to obtain asymptotic properties of the estimator (51 ).

## Applying sequential adaptive designs to select PSUs in cluster sampling

An interesting application of sequential adaptive designs is to select primary sampling units (PSU) in multi-stage cluster sampling, which is the case in our motivational tuberculosis example described in Sect. 2. We will highlight how sequential adaptive designs, and in particular PoSA and CPoSA, offer a flexible framework to manage logistics and survey budget and, at the same time, improve case detection and provide unbiased estimates of national TB prevalence. To implement TB prevalence surveys, the sampling design currently suggested by the WHO guidelines implies the selection of national sub-areas as PSUs. Sub-areas are groups of individuals purposefully informed according to specific criteria described in the guidelines book ( WHO 2011), and all individuals included in the selected PSUs are invited to participate to the survey for data collection. The spatial pattern in the surveyed country is intended on the basis of TB being an infectious disease. The one-dimensional simplification may be a pre-designed route on a geographical map that needs to be followed across the country by the field team and equipment (*e.g.* medical staff and X-ray). From a theoretical viewpoint, the choice of a route requires to create a function that maps a two-dimensional space into a one-dimensional space. This problem is also related to the inclusion of spatial aspects into the sampling design, in particular to spread the sample. A widely used methodology is the Generalized Random Tessellation Stratification (GRTS); cfr. Stevens and Olsen (1999), Stevens and Olsen (2004). A recent contribution, based on the traveling salesman problem, is in Dickson and Tillé (2016). For the purposes of our TB example, the choice of the route may be part of the survey design and may be tailored upon specific requirements and/or physical features of the country. It can be negotiated with local authorities and compromised to budget constraints. For instance, the route can be defined by minimising travel costs while at the same time acknowledging the presence of limited access areas. PSUs are to be informed along the chosen route in such a way that they result pre-ordered. Thus, the same closeness notion prescribed by both PoSA and CPoSA applies to pairs of strictly subsequent PSUs. Let $h=1 , \, \dots , \, M$ denote the ordered sequence of PSUs, and let $N_h$ be the number of individuals included in the *h*th PSU, so that $\sum _{h=1}^M N_h$ is the size of the surveyed population. The updating matrix, *i.e.* the operative device given in (1) would list the *M* selection steps on the rows, along the assigned route, and the *M* PSUs on the columns with chosen initial probabilities $\pi _h^{(0)}, \ h=1 \dots M$. At step *h*, the *h*-th PSU is visited and selected/not selected upon a Bernoulli trial with probability $\pi _{h}^{(h-1)}$ updated at the previous step. If PSU *h* is selected, all individuals are enrolled for data collection so that the actual prevalence of the PSU’s TB cases can be observed

48 $$\begin{aligned} {\bar{Y}}_h = \frac{1}{N_h}\sum _{i=1}^{N_h} y_i \end{aligned}$$

where $y_i$ is equal to 1 if unit *i* is a TB positive case, and 0 otherwise.

Upon recording the realization of PSU’s sample membership indicator $S_h=s_h$ (either 0 or 1) on the diagonal of the updating matrix, the selection probability of all subsequent PSUs $j \ge h+1$ is updated as follows. Let *t* be a chosen threshold, *e.g.* an anticipated guess or a previous estimate of the national prevalence. As condition *D* we consider here the exceedance of threshold *t*, namely

$$\begin{aligned} d_h = I_{({\bar{Y}}_h \ge t)} = \left\{ \begin{array}{c} 1 \; {\textrm{if}} \; {\bar{Y}}_h \ge t \\ 0 \; {\textrm{if}} \; {\bar{Y}}_h < t \end{array} \right. . \end{aligned}$$

In general, the updating rule is

49 $$\begin{aligned} \pi _{j}^{(h)} = \left\{ \begin{array}{ll} 1 \quad \quad \text {if } j=h+1 \ \text {and} \ S_h d_h =1 &{} \\ \pi _{j}^{(h-1)} - \left( S_h d_h - \pi _{h}^{(h-1)} \right) w^{(h)}_{j-h} &{} \text {otherwise} \end{array} \right. \end{aligned}$$

where, in particular,

50 $$\begin{aligned} w^{(h)}_{j-h}= & {} 0 \;\; {\mathrm {under \; PoSA}} \\ w^{(h)}_{j-h}= & {} \frac{1}{M-h} \;\; {\mathrm {under \; CPoSA}} . \nonumber \end{aligned}$$

The threshold *t* may also depend on *h* according to clinical reasons, logistic convenience or relevant PSU features, for instance whether rural or urban. Based on the input from the updating matrix and the sub-area prevalences computed from selected PSUs, the Pseudo-HT estimator of the overall national prevalence is given by

51 $$\begin{aligned} \widehat{{\bar{Y}}}_{PHT}= \frac{1}{N}\sum _{h=1}^M N_h {\bar{Y}}_h \frac{S_h}{\pi ^{(h-1)}_{h}} \end{aligned}$$

For variance estimation, Eq. (22) can be adapted accordingly.

We conclude with two practical comments. First of all, CPoSA can be applied by choosing a number $m_{min}$ of PSUs as minimum first-stage sample size. Consequently, the minimum size $n_{min}$ of the final sample of individuals can be planned nearby $m_{min} \cdot {\bar{N}}$, where ${\bar{N}}$ is the PSUs’ average size. WHO’s guidelines recommend designing PSUs of equal size. For instance, in Kenya’s 2015-2016 prevalence survey (Ministry of Health, Republic of Kenya, 2016), PSUs were defined as an average of 500 households, ranging between 400 and 600 households with 720 (range: 650-790) eligible people per PSU invited to participate, which ultimately led to a planned sample of $72\, 000$ individuals. However, as the variability among PSU sizes increases so does the uncertainty around the choice of $m_{min}$ and the forecast on $n_{min}$, which can limit the effectiveness of CPoSA versus PoSA in controlling the size of the final sample.

In the second place, it is worth mentioning that 100% participation of all the invited units included in the selected PSUs may harldy be the case in practice. Indeed, WHO guidelines recommend to increase the planned sample size to allow for non-participation in the survey of eligible individuals on the basis of the expected quote of participation. On the other hand, evidence of reasonable to large participation can be found in national prevalence surveys conducted in different world settings. For instance, the already mentioned Kenya 2015-2016 survey registered an 83% participation rate (87% female, 77% male, expected 85%).

## Empirical evidence

This section aims to present empirical evidence of the strengths and weaknesses of our proposed sampling strategies. A simulation has been designed in the framework of our motivational example (Sect. 6), by using the WHO guidelines and Kenya 2016 TB prevalence survey as reference model. The study has three main goals

- to explore the performance of CPoSA versus PoSA, particularly in terms of final sample size and over-sampling of positive cases;
- to investigate the performance of CPoSA versus a traditional cross-sectional, non-adaptive sampling design, based on a real application; and
- to compare the efficiency of the pseudo-HT estimator (19) w.r.t. the traditional HT estimator.

### Simulation protocol

**Fig. 1:**
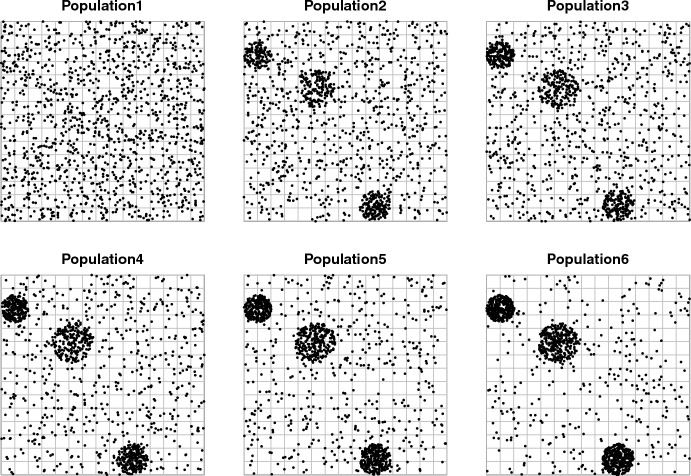
Six simulated scenarios: dots depict positive cases gathered in 3 clusters

The simulation protocol is based on a population of size $N=250\,000$ individual units over a country area, represented as a square (see Fig. 1). *y* values are generated from a Bernoulli distribution with parameter 0.005, leading to a simulated true prevalence of 0.5%. This choice is intended to simulate a rare disease and is driven by the estimated 558 cases per $100\,000$ population units from the Kenya survey. $M=225$ sub-areas (PSUs) were generated via a super-imposed $15\times 15$ grid of squares with size $N_h$ between 1034 and 1208 elementary units. According to the WHO guidelines, sampling concerns PSUs and all elementary units included into selected PSUs are included in the final sample. In particular, Pareto sampling (cfr. Rosén 1997) has been used for probability-proportional-to-size selection of PSUs and as traditional design for the purposes of comparison. Sample size for the traditional sampling design was computed according to the WHO guidelines for the recommended level of precision (less than 25% absolute error at 95% confidence level) and based on a preliminary guess of the to-be-estimated prevalence. Note that we used the true 0.005 prevalence as the anticipated guess so that the traditional design, which we shall use as benchmark for the purposes of our study, is in fact simulated in its best scenario. This choice led to plan the selection of $m_{WHO}=27$ PSUs, with forecasted $n_{WHO}=29\,290$ elementary units. The same true prevalence 0.005 has been used for both PoSA and CPoSA as threshold *t* to set the adaptive updating rule in Eq. (49). For comparisons with CPoSA, and considering its over-sampling vocation, we used $n_{WHO}$ as an upper limit to set the CPoSA minimum size of the sample of units, in the range $0.7n_{WHO} \le n_{min} \le n_{WHO}$, which led to set $m_{min}$ between 19 and 22 as minimum size for PSU selection. The required initial ordering was chosen as an up-and-down path across all 225 PSUs. This choice is intended to ensure comparability of the surveyed population with respect to the traditional design. On the other hand, such a choice may not favour either PoSA or CPoSA against the traditional non adaptive design. Other more favorable choices would be possible, for instance when auxiliary information is available that can facilitate the crossing of sub-areas with the highest clusterisation of positive cases.

Inclusion probabilities under the traditional design have been set as proportional to the PSU size $N_h$ according to the WHO guidelines, resulting in $0.11 \le m_{WHO}N_h/N \le 0.13$. The same set of inclusion probabilities has been used as initial $\pi _h^{(0)}$ under PoSA and CPoSA.

The key simulation factor is the level of spatial clustering of the study variable, i.e. how concentrated or else how spread-out are the TB cases over the surveyed region. We then generated 6 scenarios with increasing proportion of positive cases (in the range 0 to 70%) gathered in 2, 3 and 4 clusters. As a measure of such clustering we considered the coefficient of intra-area variation, according to the WHO guidelines. In particular, the coefficient of intra-area variation is defined as $k = \sqrt{V\left( {\bar{Y}}_h\right) }/{{\bar{Y}}}$, where $V\left( {\bar{Y}}_h\right) = (1/N)\sum _{h=1}^M \left( {\bar{Y}}_h - {\bar{Y}}\right) ^2 N_h$, namely the between-area variability of the study variable. Every simulation is based on 5000 Monte Carlo (MC) runs.

**Table 1:** Key features of the six simulated scenarios with 3 clusters

Pop	1	2	3	4	5	6
% of cases						
gathered into	0%	30%	40%	47%	60%	70%
the 3 clusters						
k	0.5	1.1	1.4	1.7	2.0	2.5

We focus on the 6 scenarios with 3 clusters depicted in Fig.  1 for increasing levels of clusterisation as given in Table  1. Simulations results for 3 clusters appear suitable for showing general trends. Moreover, setting 3 clusters in the population allows for simulating the widest range of clustering as given by $0.5 \le k \le 2.5$. Of course, the main impact of varying the number of clusters in the population occurs upon the final sample size of both PoSA and CPoSA, which increases as the number of clusters increases. However, aside from this effect naturally related to the adaptive component, no peculiarities have emerged, and simulation results for 3 clusters are quite uniformly intermediate between scenarios with 2 and 4 clusters.

### Comparison of PoSA and CPoSA designs

**Fig. 2:**
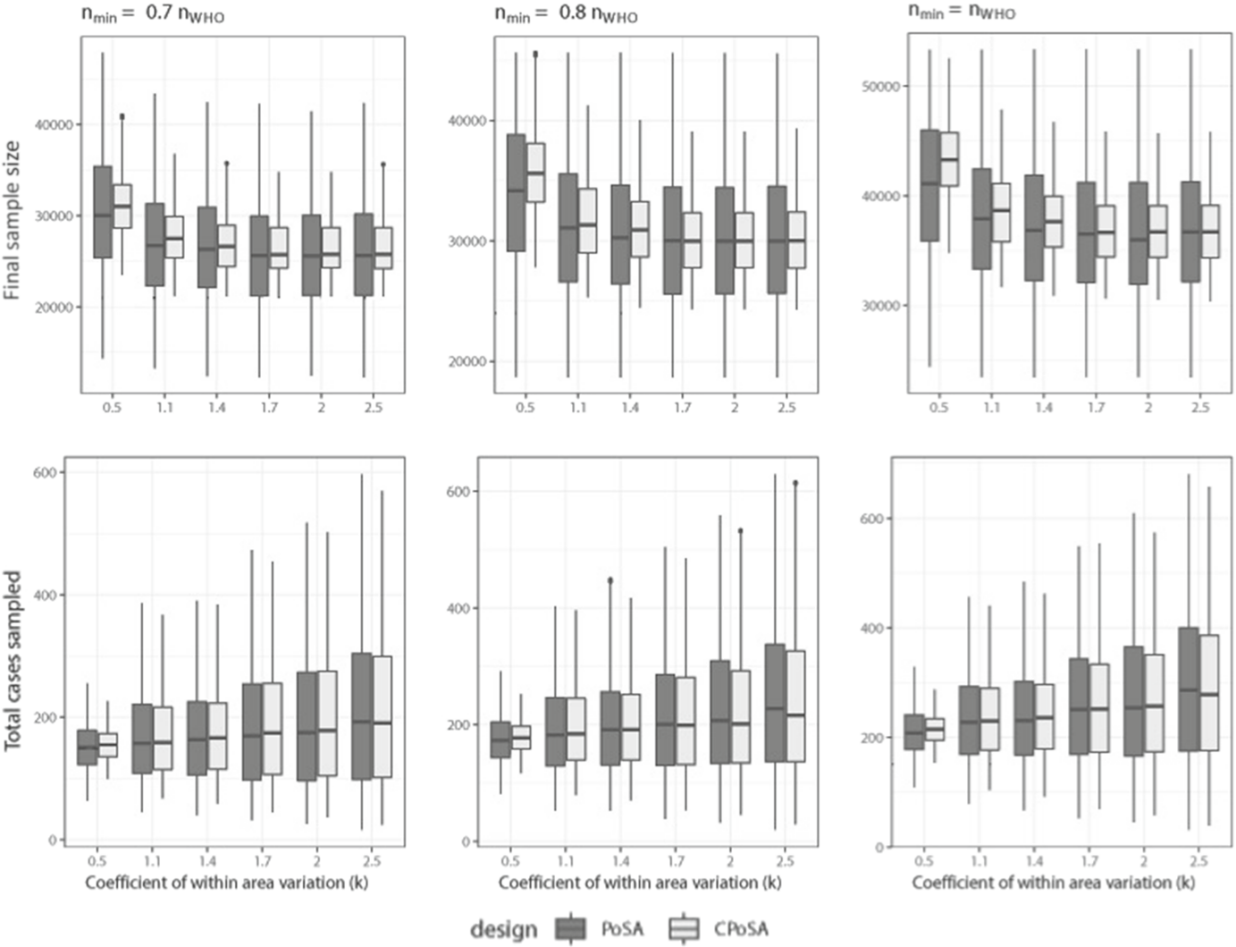
CPoSA versus PoSA for increasing values of $n_{min}$: boxplots of the MC distribution of final sample size (upper panels) and number of positive cases detected (lower panel) (98% values represented)

The performances of PoSA and CPoSA are compared with respect to two key features: the size of the provided sample and the number of positive cases selected. Results are shown in Fig. 2, where Monte Carlo (MC) distributions are plotted for increasing level of clustering (as measured by *k*) and increasing CPoSA minimum sample size. The upper panel graphs clarify how CPoSA can control sample size. It can reduce both the variability in the size of samples produced by PoSA and its outliers, i.e. excessively small and large samples. At the same time, lower panel graphs show that the ability to over-sampling positive cases is quite equally enforced by CPoSA and PoSA. Meanwhile, the two sampling designs show similar behaviour on average for an increasing level of *k* and for different values of $n_{min}$.

### Comparison of CPoSA to traditional, non-adaptive design

To give a better idea of the performance of CPoSA, in the subsequent two sets of graphs CPoSA is compared to the traditional design for 3 values of $n_{min}$ up to $n_{WHO}$ (column-wise) and for 4 relevant features (row-wise): 1) the ability to detect positive cases, as measured by the MC Expectation of the rate of positive cases into the simulated samples; 2) the accuracy of the final estimate, as measured by the MC Root Mean Squared Error of the estimator $\sqrt{E_{MC} \left( \hat{{\bar{Y}}} - {\bar{Y}} \right) ^2 }$; 3) the final sample size; and 4) the cost per case detected, as measured by the MC Expectation, across all simulated sample, of the ratio of the total survey cost over the number of positive cases selected. The survey cost has been computed under a conventional linear cost function $C=c_0 + c_1 m + \sum _{{h \in s_m}} c_2 N_{h}$ where *m* denotes the size of the sample $s_m$ of selected PSUs under a given design. The costs have been set according to the budget of the 2016 Kenya survey. In details we set fixed costs $c_0=2\,900\,000$ USD, including for instance procurement capital, training and launchs; unitary cost per PSU $c_1=18\,900$ USD, which includes cluster budget, transport and development of maps; unitary cost per elementary unit $c_2= 6.5$ USD for individual specimens processing, laboratory field expenses and consumables. A fixed $20\%$ discount has been applied to PSU cost $c_1$ under CPoSA. This choice is intended to simulate the expected savings following from the increased control over logistics and budget allocation, *e.g.* the planning of a route for sequential selection by minimising travel costs. To facilitate comparisons, all simulation results are presented for CPoSA as a ratio relative to the traditional design. Equal performance is indicated by the dashed line, while gains (losses) show above (below) the equality line.

**Fig. 3:**
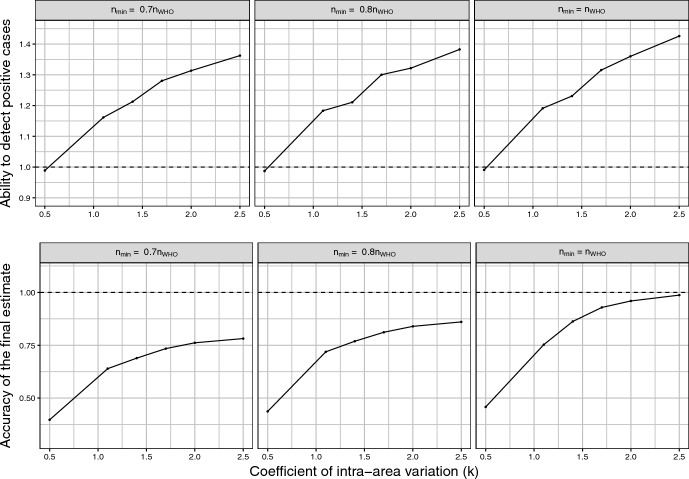
CPoSA versus traditional WHO design: over-sampling of positive cases and accuracy of the final estimate, for increasing level of *k*. Ratio over the traditional design (dashed line means equal performance)

Simulation results indicate that CPoSA improves uniformly, under all aspects explored, as the spatial clustering of positive cases increases. In particular, the CPoSA’s potential to over-sample positive cases, shown in Fig. 3 upper panels, rapidly outperforms the traditional sampling design as *k* increases from scenario of no clustering $(k=0.5)$ to 70% of clustering of positive cases $(k=2.5)$. The number of positive cases detected under CPoSA can be 1.3 to 1.45 times larger than the traditional (WHO) design, as the minimum sample size increases up to the same WHO size. The ability to over-sample positive cases that characterises CPoSA has a cost in terms of efficiency of the estimate released (Fig. 3, lower panels). The efficiency loss under CPoSA against the benchmark design can be as much as 60 to 75% in the worst-case scenario with no spatial clustering. However, CPoSA’s efficiency promptly improves as *k* increases, reducing the loss to 25-20% for higher values of spatial variability. In this case, equal efficiency is approached for larger sample size.

**Fig. 4:**
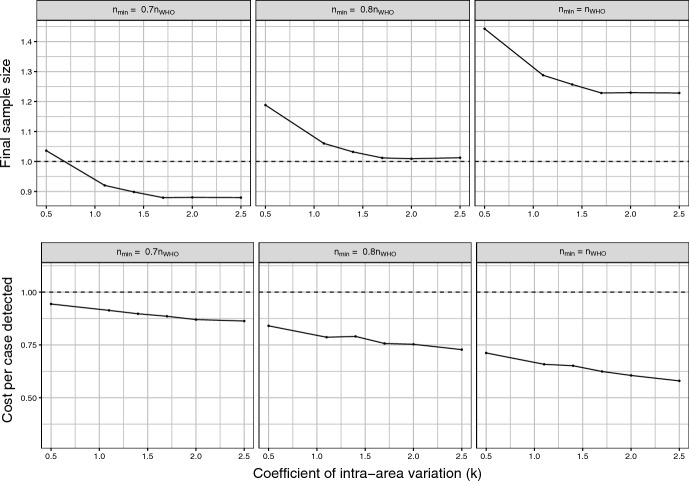
CPoSA versus traditional WHO design: final sample size and cost per case detected, for increasing level of *k*. Ratio over the traditional design (dashed line means equal performance)

Simulation results presented in the upper panels of Fig. 4 show that the size of the final sample under CPoSA can be significantly larger than the fixed size $n_{WHO}$ under the traditional design, up to 45% larger in the worst scenario of no spatial clustering $(k=0.5)$. Meanwhile, CPoSA’s final sample size rapidly reduces as *k* increases, quickly approaching the same size under the (fixed-size) traditional design when $n_{min}$ is chosen to be slightly smaller. This suggests that the planning of $n_{min}$ is a key factor to control the final sample size under CPoSA, which should be carefully balanced versus the expected level of spatial clustering. Finally, in the lower panels of Fig. 4 simulation results indicate that CPoSA outperforms the traditional sampling strategy in terms of the cost per case detected, as a mirror-effect of the over-sampling capacity, combined with the savings allowed, at the survey design phase, with regard to the logistics and PSUs budget. Again, the cost per case decreases as *k* increases. The downsize is emphasised for larger sample size, 40% less in the most clustered scenario.

### Comparison of Pseudo-HT to HT estimator

The goal of this last section is to compare the performance of the proposed pseudo-HT estimator (19) versus the traditional HT estimator, expressed in terms of estimator’s variance. It is important to remark that traditional HT estimator does not actually apply in practice, because (marginal) first order inclusion probabilities $\pi _i$ are unknown, depending on unobserved population values $y_i$s for all adaptive sampling designs considered in the present paper (cfr. Eq. 6). Heuristically, the HT estimator might be expected to be more efficient than the Pseudo-HT estimator, being based on extra-sample information, namely the unavailable $\pi _i$s for all population units. However, simulation results for the six scenarios in Fig. 1 suggest for this intuition to be false. First and second order inclusion probabilities $\pi _i$ and $\pi _{ij}$ have been computed under PoSA design, and then used to compute the exact variance $V( \widehat{\bar{Y}}_{HT} )$ of the traditional HT estimator. Finally, the exact variance of the Pseudo-HT estimator $V( \widehat{\bar{Y}}_{PoSA} )$ has been computed under the same PoSA deisgn and for the same six populations. Results are presented in Table 2.

**Table 2:** Comparing efficiency of Psudo-HT estimator vs HT estimator, under PoSA design and 6 populations (see Table 1 and Fig. 1)

Pop	$V\left( \hat{{\bar{Y}}}_{PoSA}\right)$	$V\left( \hat{{\bar{Y}}}_{HT} \right)$	Ratio
1	1.43	1.89	0.76
2	2.56	3.20	0.80
3	3.17	3.87	0.82
4	4.47	5.63	0.79
5	5.40	6.87	0.79
6	7.35	9.04	0.81

It is apparent, at least in the simulated scenarios, that Pseudo-HT estimator outperforms HT estimator under PoSA, uniformily for different levels of spatial clustering of positive cases. This is of course a nice feature of the Pseudo-HT estimator, and a solid argument in favour of its use. A possible explanation of results in Table 2 is that the final sample size under CPoSA is less variable than the PoSA design case.

## Concluding remarks

In this paper a novel class of sequential adaptive sampling strategies has been proposed, that apply to population-based surveys for a clustered study variable, as for instance an infectious disease. The underlying idea is to integrate an adaptive component into a list-sequential selection, which implies both practical advantages and methodological challenges. On the practical side, the proposed strategy allows for oversampling positive cases of the study variable while pursuing cost effectiveness. On the methodological side the informative nature of the sampling design has required the development of a proper weighting system and the construction of a class of unbiased Pseudo-HT estimators, for which consistency and asymptotic Normal distribution have been also proved. As first proposals based on Poisson sampling, two special members of the proposed class of sampling strategies, called PoSA and CPoSA, have been given special attention. However, due to their simplicity, both PoSA and CPoSA have limitations, which in fact open future research opportunities. In particular, the assumption of a linearly ordered population can be feasible and effective in the practice of TB prevalence surveys, but can reveal too strong and less viable in different application contexts. Thus, a natural improvement would be to relax the assumption of a linearly ordered population, e.g. the pre-fixed path in the TB example, to allow a two-dimensional selection that is able to move freely all along the geographical area of interest, while controlling covariances between SMIs. This could be done by leaving the Poisson list-sequential choice in favour of a more flexible design such as, for instance, the spatially correlated Poisson sampling (Grafström 2012). Future research will also explore the availability of auxiliary variable(s) and accessible paradata that can be effectively employed both at the design stage and at the estimation stage of the survey, to improve estimation accuracy. For instance, in the TB example, epidemiological, socio-cultural and/or economic covariates may be available from previous surveys and official registers. They can be exploited for an advanced definition of neighbourhood conditions either for population units or PSUs, thus refining the mere physical/geographical proximity applied to PoSA and CPoSA.

## APPENDIX: Proofs and auxiliary results

### Proof of Proposition 1

Eqns. (6 ), (7 ) are easily proved by induction. Equation ( 6 ) is true if $i=1$, because it gives $\pi _1 = \pi ^{(0)}_1$. Assuming than it holds for unit *i*, we must prove it also holds for unit $i+1$. Using the updating rule, we have

$$\begin{aligned} \pi _{i+1}= & {} E [ E [ S_{i+1} \vert \mathcal {F}_{i} ]] = E [ d_{i} S_{i} + \pi ^{(0)}_{i+1} ( 1- d_i S_i ) ] \\= & {} \pi ^{(0)}_{i+1} + \pi _i d_{i} (1- \pi ^{(0)}_{i+1} ) = \pi ^{(0)}_{i+1} + d_{i} (1- \pi ^{(0)}_{i+1} ) \left\{ \pi _{i}^{(0)} \right. \\{} & {} \left. + \sum _{j=1}^{i-1} \pi _j^{(0)} \prod _{h=j+1}^{i} \left( 1-\pi _h^{(0)}\right) d_{h-1} \right\} \\= & {} \pi _{i+1}^{(0)} + \sum _{j=1}^{i} \pi _j^{(0)} \prod _{h=j+1}^{i+1} \left( 1-\pi _h^{(0)}\right) d_{h-1} . \end{aligned}$$

Equation ( 7 ) is proved similarly. It is easy to verify that it is true as $k=1$, because

$$\begin{aligned} \pi _{i, i+1}= & {} E [ S_i S_{i+1} ] = E [ S_{i} E[ S_{i+1} \vert \mathcal {F}_{i} ]] \\= & {} E[ S_{i} ( \pi ^{(0)}_{i+1}+ S_i d_i (1- \pi ^{(0)}_{i+1} ) ] = E[ S_{i} ( \pi ^{(0)}_{i+1} + d_i ( 1- \pi ^{(0)}_{i+1} ) ) ] \\= & {} \pi _i ( \pi ^{(0)}_{i+1} + d_i ( 1- \pi ^{(0)}_{i+1} ) ) \end{aligned}$$

coincides with ( 7 ) for $k=1$. Assuming next that ( 7 ) holds for *k*, we have

$$\begin{aligned} \pi _{i , i+k+1}= & {} E [ S_i E [ S_{i+k+1} \vert \mathcal {F}_{i+k} ]] = E[ S_i ( \pi ^{(0)}_{i+k+1} + S_{i+k} d_{i+k} (1- \pi ^{(0)}_{i+k+1} ) ] \\= & {} \pi _i \pi ^{(0)}_{i+k+1} + d_{i+k} (1- \pi ^{(0)}_{i+k+1} ) \pi _{i , i+k} \\= & {} \pi _i \pi ^{(0)}_{i+k+1} + d_{i+k} (1- \pi ^{(0)}_{i+k+1} ) \left\{ \pi _i \left( \pi ^{(0)}_{i+k} + \sum _{j=i+1}^{i+k-1} \pi _j^{(0)} \prod _{h=j}^{i+k-1} (1- \pi ^{(0)}_{h+1} ) d_h \right. \right. \\&\,&\left. \left. + \prod _{h=i}^{i+k-1} (1- \pi ^{(0)}_{h+1} ) d_h \right) \right\} \\= & {} \pi _i \left\{ \pi ^{(0)}_{i+k+1} + \sum _{j=i+1}^{i+k} \pi _j^{(0)} \prod _{h=j}^{i+k} (1- \pi ^{(0)}_{h+1} ) d_h + \prod _{h=i}^{i+k} (1- \pi ^{(0)}_{h+1} ) d_h \right\} . \end{aligned}$$

$$\square$$

### Proof of Proposition 2

The proof is immediate by observing that

52 $$\begin{aligned} E \left[ T_{i_{1}} T_{i_{2}} \cdots T_{i_{n}} \right]= & {} E \left[ E \left[ T_{i_{1}} T_{i_{2}} \cdots T_{i_{n}} \vert \mathcal {F}_{i_{n} -1} \right] \right] \nonumber \\= & {} E \left[ T_{i_{1}} T_{i_{2}} \cdots T_{i_{n-1}} E \left[ T_{i_{n}} \vert \mathcal {F}_{i_{n} -1} \right] \right] \nonumber \\= & {} 0 \end{aligned}$$

because $E \left[ T_{i_{n}} \vert \mathcal {F}_{i_{n} -1} \right] =0$ in view of ( 3 ). $\square$

### Proof of Proposition 3

Unbiasedness of $\widehat{{\bar{Y}}}_{PHT}$ is an immediate consequence of $E[ T_i ] =0$. As far as its variance is concerned, since $T_i$s are pair-wise uncorrelated we have first

$$\begin{aligned} V \left( \widehat{{\bar{Y}}}_{PHT} \right) = \frac{1}{N^2} \sum _{i=1}^{N} V \left( \frac{S_i}{\pi _i^{(i-1)}} \right) y_i^2 . \end{aligned}$$

Next, using ( 3 ) and taking into account that

$$\begin{aligned} E \left[ \left. \frac{S_i}{\pi _i^{(i-1)}} \right| \mathcal {F}_{i-1} \right] = 1 \end{aligned}$$

we have

$$\begin{aligned} V \left( \frac{S_i}{\pi _i^{(i-1)}} \right)= & {} E \left[ V \left( \left. \frac{S_i}{\pi _i^{(i-1)}} \right| \mathcal {F}_{i-1} \right) \right] + V \left( E \left[ \left. \frac{S_i}{\pi _i^{(i-1)}} \right| \mathcal {F}_{i-1} \right] \right) \\= & {} E \left[ \left( \frac{1}{\pi _i^{(i-1)}} \right) ^2 V \left( \left. S_i \right| \mathcal {F}_{i-1} \right) \right] \\= & {} E \left[ \left( \frac{1}{\pi _i^{(i-1)}} \right) ^2 \pi _i^{(i-1)} (1- \pi _i^{(i-1)} ) \right] \end{aligned}$$

from which Eq. ( 21 ) follows. $\square$

### Proof of Proposition 4

In view of Proposition 3, it is enough to prove that the variance of $\widehat{{\bar{Y}}}_{PoSA}$ tends to 0 as *N* increases. From $\pi ^{(i-1)}_{i} \ge \pi ^{(0)}_i \ge \delta _N$ it is immediate to see that

$$\begin{aligned} E \left[ \frac{1}{\pi ^{(i-1)}_{i}} \right] \le \frac{1}{\delta _{N}} \end{aligned}$$

and hence

$$\begin{aligned} V \left( \widehat{{\bar{Y}}}_{PoSA} \right) \le \frac{1}{N^{2}} \sum _{i=1}^{N} \left( \frac{1}{\delta _{N}} -1 \right) y_i^2 \le \frac{M^{2}}{N \delta _{N}} \rightarrow 0 \end{aligned}$$

from which ( 28 ) follows. $\square$

### Proof of Lemma 1

In the first place, from $E[ ( S_i / \pi ^{(i-1)}_{i} -1 )^2 \vert \mathcal {F}_{i-1} ] = 1 / \pi ^{(i-1)}_{i} -1$ we get

53 $$\begin{aligned} E \left[ \frac{1}{N} \sum _{i=1}^{N} E[ X_{Ni}^2 \vert \mathcal {F}_{i-1} ] \right] = 1 \end{aligned}$$

for every $$N \ge 1$$.

In the second place, from ( 23 ) we may write

54 $$\begin{aligned}&\,&V \left( \frac{1}{N} \sum _{i=1}^{N} E[ X_{Ni}^2 \vert \mathcal {F}_{i-1} ] \right) = V \left( \frac{1}{\sigma _{N}^{2}} \sum _{i=1}^{N} \left( \frac{1}{\pi ^{(0)}_i} -1 \right) (1- S_{i-1} d_{i-1} ) y^2_i \right) \nonumber \\&\,&= \frac{1}{\sigma ^{4}_{N}} \sum _{i=1}^{N} \left( \frac{1}{\pi ^{(0)}_i} -1 \right) ^2 y_i^4 d_{i-1} V( S_{i-1} ) \nonumber \\&\,&+ \frac{2}{\sigma ^{4}_{N}} \sum _{i=1}^{N} \sum _{j=i+1}^{N} \left( \frac{1}{\pi ^{(0)}_i} -1 \right) \left( \frac{1}{\pi ^{(0)}_j} -1 \right) y^2_i y^2_j d_{i-1} d_{j-1} C( S_{i-1} , \, S_{j-1} ) \;\; \end{aligned}$$

where $C( S_{i-1} , \, S_{j-1} )$ is the covariance between $S_{i-1}$ and $S_{j-1}$.

Now, from $V ( S_{i-1} ) = \pi _{i-1} ( 1- \pi _{i-1} ) \le 1/4$ and $1/ \pi ^{(0)}_i \le \delta ^{-1}$ we have

55 $$\begin{aligned} \frac{1}{\sigma ^{4}_{N}} \sum _{i=1}^{N} \left( \frac{1}{\pi ^{(0)}_i} -1 \right) ^2 y_i^4 d_{i-1} V( S_{i-1} )\le & {} \frac{1}{\sigma ^{4}_{N}} \sum _{i=1}^{N} \left( \frac{1}{\pi ^{(0)}_i} -1 \right) ^2 y_i^4 \nonumber \\\le & {} \frac{M^4}{4 c^2 N} \left( \frac{1}{\delta } -1 \right) ^2 \rightarrow 0 \end{aligned}$$

ad *N* increases.

As a consequence of ( 8 ) and subsequent remarks, the covariance between $S_{i-1}$ and $S_{j-1}$ is non-zero only in two cases: (*a*) both units $i-1$ and $j-1$ lie in the same block $B^1_k$; (*ii*) $(i-1) \in B^1_k$ and $j-1$ is the first unit of the block $B^0_{k+1}$, for some $k=1, \, \dots , \, K$. Denote again by $i_{k+1 , 0}$ the first unit of the block $B^0_{k+1}$, and by $B^{1+}_k$ the set $B^1_k \bigcup \{ i_{k+1 , 0} \}$, and by $\vert B^{t}_k \vert$ the cardinality of the set $B^t_k$, $t=0, \, 1$. Since $C( S_i , \, S_j ) \le 1/4$, we have

56 $$\begin{aligned}&\,&\frac{2}{\sigma ^{4}_{N}} \sum _{i=1}^{N} \sum _{j=i+1}^{N} \left( \frac{1}{\pi ^{(0)}_i} -1 \right) \left( \frac{1}{\pi ^{(0)}_j} -1 \right) y^2_i y^2_j d_{i-1} d_{j-1} C( S_{i-1} , \, S_{j-1} ) \nonumber \\\le & {} \frac{1}{2 \sigma ^{4}_{N}} \sum _{k=1}^{K} \sum _{i \in B^{1+}_{k}} \sum _{1 < j \in B^{1+}_{k}} \left( \frac{1}{\pi ^{(0)}_i} -1 \right) \left( \frac{1}{\pi ^{(0)}_j} -1 \right) y^2_i y^2_j \vert B^{1+}_{k} + 1 \vert ^2 \nonumber \\\le & {} \frac{M^{4}}{2 c^{2} N^{2}} \left( \frac{1}{\delta } + 1 \right) ^2 K \max _{1 \le k \le K} \vert B^{1+}_{k} + 1 \vert ^2 . \nonumber \\\rightarrow & {} 0 \;\; {\textrm{as}} \; N \rightarrow \infty \end{aligned}$$

by Assumption A5.

The proof now follows from ( 53 )-( 56 ). $\square$

### Proof of Lemma 2

From $\pi ^{(i-1)}_i \ge \pi ^{(0)}_i \ge \delta$ and Eq. ( 27 ), we get

57 $$\begin{aligned} E \left[ X_{Ni}^4 \right]= & {} \frac{N^2}{\sigma ^4_N} \left[ \left( \frac{S_{i}}{\pi ^{(i-1)}_{i}} - 1 \right) ^4 \right] y^4_i \le \frac{N^2}{\sigma ^4_N} M^4 \left( \frac{1}{\delta } + 1 \right) ^4 \nonumber \\\le & {} \frac{M^4}{c^2} \left( \frac{1}{\delta } + 1 \right) ^4 \;\; \forall i=1 , \, \dots , \, N \end{aligned}$$

for all *N*s large enough. The Lemma easily follows from ( 57 ). $\square$

### Proof of Proposition 5

To prove ( 32 ), it is enough to take into account that

$$\begin{aligned} \frac{\widehat{\bar{Y}}_{PoSA} - \overline{y}_N}{\sqrt{V (\widehat{\bar{Y}}_{PoSA})}} = \frac{1}{\sqrt{N}} \sum _{i=1}^{N} X_{Ni} \end{aligned}$$

and to use Lemmas 1, 2 and Th. 1.3 in Alj et al. (2014).

To prove ( 33 ), it is enough to prove that

58 $$\begin{aligned} \frac{N}{\sigma _{N}^2} \frac{1}{N} \sum _{i=1}^{N} \frac{S_{i}}{\pi ^{(i-1)}_{i}} \left( \frac{1}{\pi _{i}^{(i-1)}} - 1 \right) y_i^2 {\mathop {\rightarrow }\limits ^{p}} 1 \;\; {\textrm{as}} \; N \rightarrow \infty . \end{aligned}$$

To this purpose, observe that

$$\begin{aligned} N \widehat{V}_{PHT} = \frac{1}{N} \sum _{i=1}^{N} Y_{Ni} + \frac{1}{N} \sum _{i=1}^{N} Y^{\prime }_{Ni} + \end{aligned}$$

where

$$\begin{aligned} Y_{Ni}= & {} \left( \frac{1}{\pi _{i}^{(i-1)}} - 1 \right) y_i^2 , \\ Y_{Ni}^{\prime }= & {} \left( \frac{S_i}{\pi _{i}^{(i-1)}} - 1 \right) \left( \frac{1}{\pi _{i}^{(i-1)}} - 1 \right) y_i^2 . \end{aligned}$$

Taking into account that $E\left[ Y^{\prime }_{Ni} \vert \mathcal {F}_{i-1} \right] =0$ and, by A1, A4,

$$\begin{aligned} \limsup _{M \rightarrow \infty } \sum _{i=1}^{N} \frac{1}{i^2} E [ Y_{i,N}^2 ] < \infty \end{aligned}$$

from Csörgő (1968) it follows that

59 $$\begin{aligned} \frac{1}{N} \sum _{i=1}^{N} Y_{Ni}^{\prime } {\mathop {\rightarrow }\limits ^{p}} 0 \;\; {\textrm{as}} \; N \rightarrow \infty . \end{aligned}$$

In the second place, as an easy consequence of Lemma 1 it is seen that

60 $$\begin{aligned} \frac{N}{\sigma _{N}^{2}} \frac{1}{N} \sum _{i=1}^{N} \left( \frac{1}{\pi _{i}^{(i-1)}} - 1 \right) y_i^2 {\mathop {\rightarrow }\limits ^{p}} 1 \;\; {\textrm{as}} \; N \rightarrow \infty . \end{aligned}$$

Hence, ( 58 ) now follows from ( 59 ), ( 60 ). Finally, ( 34 ) is an immediate consequence of ( 32 ) and ( 33 ). $\square$

### Proof of Lemma 3

From the updating rule ( 2 ) it is not difficult to see that

61 $$\begin{aligned} \pi ^{(i)}_{i+1}= & {} S_i d_i + (1- S_i d_i ) \left\{ \pi ^{(i-1)}_{i+1} - w^{(i)}_{1} \left( S_i - \pi ^{(i-1)}_{i} \right) \right\} = S_i d_i + (1-S_i d_i ) \pi ^{(i-1)}_{i+1} - c_{i1} \nonumber \\= & {} S_1 d_1 + (1-S_i d_i ) \left\{ \pi ^{(i-2)}_{i+1} - w^{(i-1)}_{2} \left( S_{i-1} - \pi ^{(i-2)}_{i-1} \right) \right\} - c_{i1} \nonumber \\= & {} S_i d_i + (1-S_i d_i ) \pi ^{(i-2)}_{i+1} - ( c_{i1} + c_{i2} ) \nonumber \\&\cdots =&S_i d_i + (1- S_i d_i ) \pi ^{(0)}_{i+1} - ( c_{i1} + c_{i2} + \cdots + c_{ii} ) \nonumber \\= & {} S_i d_i + (1- S_i d_i ) \pi ^{(0)}_{i+1} - C_i \end{aligned}$$

where $c_i$s and $C_i$s are defined as in ( 37 ), ( 36 ), respectively.

To prove inequality ( 38 ), let us first observe that $( ( S_{i-k+1} - \pi ^{(i-k)}_{i-k+1} ) w^{i-k+1}_{k} ; \; k=1 , \, \dots , \, i )$ is a martingale difference w.r.t. the filtration $\mathcal {F}_{1} , \, \dots , \, \mathcal {F}_{i}$, because $E [ ( S_{i-k+1} - \pi ^{(i-k)}_{i-k+1} ) w^{i-k+1}_{k} \vert \mathcal {F}_{i-k} ] = ( E [ S_{i-k+1} \vert \mathcal {F}_{i-k} ] - \pi ^{(i-k)}_{i-k+1} ) w^{i-k+1}_{k} = 0$, $w^{i-k+1}_{k}$ being $\mathcal {F}_{i-k}$-measurable. As a consequence, the r.v.s $( S_{i-k+1} - \pi ^{(i-k)}_{i-k+1} ) w^{i-k+1}_{k}$ are pair-wise uncorrelated. Using the Lyapunov inequality, we then have

$$\begin{aligned} E \left[ \vert C_i \vert \right]\le & {} \sqrt{E \left[ C_i^2 \right] } \\= & {} \sqrt{E \left[ ( 1- S_{i-1} d_{i-1} )^2 \left\{ \sum _{k=1}^{i} \left( S_{i-k+1} - \pi ^{(i-k)}_{i-k+1} \right) w^{i-k+1}_{k} \right\} ^2 \right] } \\\le & {} \sqrt{E \left[ \left\{ \sum _{k=1}^{i} \left( S_{i-k+1} - \pi ^{(i-k)}_{i-k+1} \right) w^{i-k+1}_{k} \right\} ^2 \right] } \\\le & {} \sqrt{\sum _{k=1}^{i} E \left[ \left( S_{i-k+1} - \pi ^{(i-k)}_{i-k+1} \right) ^2 \left( w^{i-k+1}_{k} \right) ^2 \right] } \\\le & {} \sqrt{\sum _{k=1}^{i} E \left[ \left( w^{i-k+1}_{k} \right) ^2 \right] } = A_{N,i} \end{aligned}$$

because both $\vert 1- S_i d_i \vert$ and $\vert S_{i-k+1} - \pi ^{(i-k)}_{i-k+1} \vert$ are smaller than 1. $\square$

### Proof of Proposition 6

Using the same arguments as in Proposition 4, we have to show that the variance of $\widehat{\bar{Y}}_{PHT}$ tends to 0 as *N* increases. From relationship (35), we get

$$\begin{aligned} V (\widehat{\bar{Y}}_{PHT})= & {} \frac{1}{N^{2}} \sum _{i=1}^{N} E \left[ \frac{1}{\pi ^{(i-1)}_{i}} -1 \right] y^2_i \\= & {} \frac{1}{N^{2}} \sum _{i=1}^{N} E \left[ \frac{1}{\pi ^{(i-1)}_{i,PoSA}} -1 \right] y^2_i + \frac{1}{N^{2}} \sum _{i=1}^{N} E \left[ \frac{1}{\pi ^{(i-1)}_{i}} - \frac{1}{\pi ^{(i-1)}_{i,PoSA}} \right] y^2_i \\\le & {} \frac{1}{N^{2}} \sum _{i=1}^{N} \left( \frac{1}{\delta _{N}} -1 \right) y^2_i + \frac{1}{N^{2}} \left| \sum _{i=1}^{N} E \left[ \frac{C_{i-1}}{\pi ^{(i-1)}_{i} \, \pi ^{(i-1)}_{i,PoSA}} \right] y^2_i \right| \\\le & {} \frac{M^2}{N} \left( \frac{1}{\delta _{N}} -1 \right) + \frac{M^2}{N^{2}\delta _{N}^{2}} \sum _{i=1}^{N} E \left[ \vert C_{i-1} \vert \right] \\\le & {} \frac{1+ \delta _N}{N \delta } M^2 + \frac{1}{N^2 \delta _N^2} \sum _{i=1}^{N} A_{N,i-1} \\= & {} \frac{1+ \delta _N}{N \delta _N} + \frac{o(N)}{N^2 \delta _N^{2}} \rightarrow 0 \;\; {\textrm{as}} \; N \rightarrow \infty \end{aligned}$$

which proves the result. $\square$

### Proof of Lemma 4

Proof is a consequence of Lemma 3, because

$$\begin{aligned} E \left[ \left| \frac{1}{N} \sum _{i=1}^{N} \left( \frac{1}{\pi ^{(i-1)}_{i}} - \frac{1}{\pi ^{(i-1)}_{i,PoSA}} \right) y^2_i \right| \right]\le & {} \frac{M^{2}}{N \delta ^2} \sum _{i=1}^{N} E \left[ \left| C_{N,i-1} \right| \right] \\\le & {} \frac{M^{2}}{N \delta ^2} \sum _{i=1}^{N} A_{N,i-1} \rightarrow 0 \end{aligned}$$

as *N* increases, by Assumption A6. $\square$

### Proof of Lemma 5

It is enough to use Lemma 4, from which we obtain

$$\begin{aligned} E \left[ \frac{1}{\pi ^{(i-1)}_i} -1 \right]= & {} E \left[ \frac{1}{\pi ^{(i-1)}_{i,PoSA}} -1 \right] + o(1) \\ \left( \frac{S_{i}}{\pi ^{(i-1)}_{i}} -1 \right) ^4= & {} \left( \frac{S_{i}}{\pi ^{(i-1)}_{i,PoSA}} -1 \right) ^4 + o_p(1) \end{aligned}$$

and to repeat *verbatim* the arguments of Lemmas 1, 2. $\square$

### Proof of Proposition 7

It suffices to use Lemmas 5 and Th. 1.3 in Alj et al. (2014). $\square$
